# A comparison of cerebral vasomotor reactivity in diabetic and nondiabetic Iranian patients

**Published:** 2010

**Authors:** Mehdi Moghaddasi, Mansoureh Mamarabadi, Amir Hosein Habibi

**Affiliations:** a Assistant Professor of Neurology, Department of Neurology of Rasool Akram Hospital, Iran University of Medical Sciences, Tehran, Iran; b Assistant, Department of Neurology of Rasool Akram Hospital, Iran University of Medical Sciences, Tehran, Iran; c Neurologist, Ilam, Iran

**Keywords:** Vasomotor Reactivity, Transcranial Doppler, CO2 Inhalation, Diabetes Mellitus

## Abstract

**BACKGROUND:**

Cerebral microangiopathy is one of the most important complications in diabetes. It may interfere with cerebral vasomotor reactivity (VMR) which may lead to disability, stroke or even death. The aim of the present study was to determine and compare VMR changes in diabetic and non- diabetic patients.

**METHODS:**

Fifty three diabetic and 51 non- diabetic patients (with no other vascular abnormality) were recruited. Vasomotor reactivity was evaluated with Trans- Cranial Doppler (TCD) before and after CO2 inhalation.

**RESULTS:**

There were 69 (66.30%) males. The mean age was 41.53 ± 17.80 years. The general average of VMR was 5.79 ± 3.00%, the figures in diabetic and nondiabetic patients were 5.31 ± 2.60% and 6.62 ± 2.00%, respectively (p = 0.02). The average of flow velocity (FV) change was 42.47± 29.00 in diabetics and 53.34 ±16.70 in non- diabetic patients (p = 0.04).

**CONCLUSIONS:**

It is recommended that such non- invasive method is necessary for evaluation of cerebral vasculature in diabetic patients for better prevention.

Transcranial Doppler (TCD) ultrasonography is a safe, noninvasive, and low-cost technique which neurologists utilize it for imaging the large intracranial vessels at the skull base.^1^ This technique, which became available in 1982, enables assessment of hemodynamic parameters including flow velocity in intracranial arteries.^2^ The ability of the flow velocity to adapt to stimuli like hypercapnia is called vasomotor reactivity (VMR), which is another hemodynamic parameter.^3^

Cerebral blood flow is regulated by changing in arterioles resistance,^4^,^5^ i.e. with their dilatation and constriction. A profound decrease in cerebral perfusion may lead to ischemia and the vasomotor reactivity will abolish when the cerebral vessels get their maximum dilatation,^6^,^7^ indeed the vessels don’t respond to any vasodilator stimulant even to hypercapnia which is the most powerful stimulant.^8^,^9^

Diabetes is one of the well known risk factor for cerebrovascular accident (CVA).^4^,^10^ Prolonged untreated diabetes mellitus leads to microangiopathy, tissue hypoxia and ischemic lesions; it increases the risk for stroke and exacerbates brain tissue damage following ischemia.^10^,^11^ Its prevalence in diabetic patients is 2-6 times more than non- diabetics^12^–^14^ and eventually its complications and subsequent morbidity is higher, too. Patients exhibit advanced atherosclerosis in coronary and cerebral arteries as well as enhanced vascular responsiveness to vasoconstrictors, an attenuated response to vasodilators and impaired autoregulation of cerebral blood flow. Altered endothelial function of arterioles and an impaired vasomotor function of resistance vessels can contribute to altered regulation of regional blood flow and insufficient tissue perfusion in diabetes mellitus.^10^,^11^ Therefore, early diagnosis and treatment of vascular complications may lead to better care of diabetic patients and prevent consequent complications.^8^,^9^,^12^

TCD and VMR provide non-invasive and easy methods in predicting high risk patients for cerebrovascular accident,^11^ particularly to lessen the mortality and morbidity rate at least in the elective situations and predictable stresses such as surgery and other medical illnesses.^4^,^13^

The current study is conducted to determine the VMR changes in diabetic and nondiabetic patients.

## Methods

### 

#### Population

A cross-sectional study was conducted at a referral hospital affiliated to the Iran University of Medical Sciences (IUMS). Fifty-three patients with definite diagnosis of diabetes mellitus (NIDDM) who referred to endocrinology clinic were enrolled.

Controls were 51 participants selected from those who visited the orthopedic minor trauma outpatient clinic of the same hospital or persons accompanying patients who admitted to the neurology ward. Selection of both cases and controls was done by an investigator (assistant of neurology). All participants (both patients and controls) underwent TCD by one neurologist who was unaware of their status.

Non-cooperative cases and controls and those with a history of cerebrovascular diseases, coronary artery disease, hypertention, any vasculitis, anatomic abnormality of middle cerebral artery and poor window in TCD were excluded from the study.

The Medical Ethics Committee of IUMS approved the study. Written informed consent as obtained from all cases and controls before participation in the study.

#### Transcranial Doppler

This study was performed (with Explorer-CVS, DMS, France, Probe 2 MHz) to measure the cerebral blood flow velocity in the middle cerebral artery on both sides. End diastolic, peak systolic, and mean cerebral blood flow velocities were recorded automatically. After the finding of middle cerebral artery (MCA), the cerebrovascular CO2 reactivity measurement was performed as follows: the cerebral blood flow velocity was measured continuously and the participants first breathed room air through an anesthetic mask. Participants were then asked to inhale a mixture of 5% carbon dioxide in 95% oxygen for 2 minutes. The blood velocity of MCA is expected to increase after 20-30 seconds. The flow velocity (FV) indices were recorded if there was no increasing in the velocity.

The cerebral VMR was determined according to indices as below:

$$VMR\;=\;\frac{FV\;hyper-FV\;rest}{FV\;rest\;\left(Pco2\;hyper-Pco2\;rest\right)}\;\times \;100$$

#### Data Analysis

To compare the average of FV and VMR between the two groups independent t-test was utilized. A p value of < 0.05 was considered significant. All calculations were performed with the SPSS version 13.0 for Windows (SPSS Inc., Chicago, Illinois, USA).

## Results

All of 104 participants in this study were evaluated. There were 69 (66.30%) males and 35 females. The mean age (± SD) was 41.53 ± 17.80 years. The average of flow velocity (FV) changes in general was 47.80 ± 24.35 cm/s. In diabetic and non- diabetic patients the average FV were 42.47 ± 29.00 cm/s and 53.34 ± 16.70 cm/s, respectively (p = 0.04). The general average of VMR was 5.79 ± 3.00%. The figures in diabetic and nondiabetic patients were 5.31 ± 2.60% and 6.62 ± 2.00%, respectively (p = 0.02). The range of VMR in diabetic and nondiabetic cases was 1.71- 8.91% and 4.61- 8.66%, respectively. The average FV and VMR was compared between the two groups regarding age and it showed significant differences (p = 0.009). However, there was no statistical significance regarding sex (p = 0.06). The findings are summarized in tables  1 and 2

**Table 1:** The average VMR in participants regarding to gender

	Gender	Number	VMR (SD) (%)
Diabetic	M	21	5.80 (3.70)
	F	32	4.95 (3.50)
Non-diabetic	M	48	6.58 (2.00)
	F	3	7.94 (2.20)
Total	M	69	6.36 (2.60)
	F	35	5.20 (3.50)

VMR: vasomotor reactivity; M: male; F: female

**Table 2:** The average VMR and FV in diabetic and non-diabetic patients

	VMR			FV (SD) (cm/s)		
	Average (SD)	Range	P value	Average (SD)	Range	P value
Diabetic	5.31 (2.60)	5.31 ± 2.60	0.02	42.47 (29.07)	42.27 ± 29.00	0.04
Non-diabetic	6.62 (2.00)	6.62 ± 2.00		53.34 (16.70)	53.34 ± 16.70	
Total	5.79 (3.00)	5.79 ± 3.00	-	47.80 (24.35)	47.80 ± 24.35	-

FV: flow velocity (cm/s)

## Discussion

Cerebrovascular reactivity is a hemodynamic parameter representing the increase in normal cerebral artery blood flow in response to a vasodilator stimulus such as hypercapnia.^3^,^4^,^7^,^8^,^12^,^14^ An early preclinical detection of cerebrovascular complications in individuals with diabetes is one of the goals in health care.^9^ In this study the VMR or cerebral vessels reserve was evaluated. The difference between minimum and maximum of cerebral arteries diameter demonstrates the cerebral reserve and the more the difference, the more the reserve.^3^ The importance of such a reserve will be elucidated by the fact that whenever brain encounters any stress, it increases its blood volume in defiance of it, so more increase in blood flow would be more effective against stresses, although it depends on many factors such as age, gender, systemic hypertension and other vascular diseases. It is estimated that diabetes may reduce the cerebral reserve by the affect on the elasticity of the vessels.^7^,^8^,^12^ It was found in the present study that the average of VMR was 5.31 ± 2.60% in diabetics and 6.62 ± 2.00% in nondiabetic patients (p = 0.022). The present findings are compatible with a study conducted in Debercen University, Hungary, showing a significant difference between VMR in diabetic and nondiabetic patients.^9^ Also, a study performed by Lipsitz et al showed that cerebrovascular reactivity was impaired in normotensive NIDDM patients.^14^ In the present study, it was found that sex may be a factor affecting VMR, which is consistent with previous study.^9^

## Conclusions

The present results indicate that diabetes may decrease the brain blood flow reserve independent to age that may go along with an increasing risk for vascular events, although it still remains unknown if the duration of diabetes will enhance this deleterious effect. To elucidate the effect of sex, further studies with more patients may be needed.
